# Protective Effect of Fustin against Huntington’s Disease in 3-Nitropropionic Treated Rats via Downregulation of Oxidative Stress and Alteration in Neurotransmitters and Brain-Derived Neurotrophic Factor Activity

**DOI:** 10.3390/biomedicines10123021

**Published:** 2022-11-23

**Authors:** May Nasser Bin-Jumah, Sadaf Jamal Gilani, Abdulaziz F. Alabbasi, Fahad A. Al-Abbasi, Shareefa A. AlGhamdi, Ohoud Y. Alshehri, Amira M. Alghamdi, Nadeem Sayyed, Imran Kazmi

**Affiliations:** 1 Biology Department, College of Science, Princess Nourah bint Abdulrahman University, Riyadh 11671, Saudi Arabia; 2 Environment and Biomaterial Unit, Health Sciences Research Center, Princess Nourah bint Abdulrahman University, Riyadh 11671, Saudi Arabia; 3 Saudi Society for Applied Science, Princess Nourah bint Abdulrahman University, Riyadh 11671, Saudi Arabia; 4 Department of Basic Health Sciences, Preparatory Year, Princess Nourah bint Abdulrahman University, Riyadh 11671, Saudi Arabia; 5 Faculty of Medicine, King Abdulaziz University, Jeddah 21589, Saudi Arabia; 6 Department of Biochemistry, Faculty of Sciences, King Abdulaziz University, Jeddah 21589, Saudi Arabia; 7 Experimental Biochemistry Unit, King Fahd Medical Research Center, King Abdulaziz University, Jeddah 21589, Saudi Arabia; 8 Department of Biochemistry, College of Medicine, Al-Imam Mohammad Ibn Saud Islamic University (IMSIU), Riyadh 11564, Saudi Arabia; 9 School of Pharmacy, Glocal University, Saharanpur 247121, Uttar Pradesh, India

**Keywords:** 3-nitropropionic acid, Huntington’s disease, fustin, neuroprotection, tumor necrosis factor-α, brain-derived neurotrophic factor

## Abstract

Researchers have revealed that *Rhus verniciflua* heartwood, which contains fustin as an important component, possesses antioxidant-mediated, anti-mutagenic, and anti-rheumatoid arthritis characteristics. Additionally, out of the numerous plant-derived secondary metabolites, there are various research papers concentrating on flavonoids for potential advantages in neurological illnesses. The current study aims to assess the neuroprotective potential of fustin in rodents over 3-nitropropionic acid (3-NPA)-induced Huntington’s disease (HD)-like consequences. The efficacy of fustin 50 and 100 mg/kg was studied with multiple-dose administrations of 3-NPA, which experimentally induced HD-like symptoms in rats for 22 days. At the end of the study, several behavioral tests were performed including a beam walk, rotarod, and grip strength tests. Similarly, some biochemical parameters were assessed to support oxidative stress (reduced glutathione—GSH, superoxide dismutase—SOD, catalase—CAT, and malondialdehyde—MDA), alteration in neurotransmitters (gamma-aminobutyric acid—GABA—and glutamate), alteration in brain-derived neurotrophic factor activity, and nitrite levels. Additionally, pro-inflammatory parameters were carried out to evaluate the neuroinflammatory responses associated with streptozotocin such as TNF-α, IL-1β, and COX in the perfused brain. The fustin-treated group exhibited a significant restoration of memory function via modulation in behavioral activities. Moreover, 3-NPA altered biochemical, neurotransmitters, brain protein levels, and neuroinflammatory measures, which fustin efficiently restored. This is the first report demonstrating the efficacy of novel phytoconstituent fustin as a potential future candidate for the treatment of HD via offering neuroprotection by subsiding the oxidative and enzymatic activity in the 3-NPA experimental animal paradigm.

## 1. Introduction

Degeneration of neurons occurs when neurons lose their structure or function over time [1]. Neurodegenerative proteinopathies are caused by a malfunctioning protein in the brain. Essentially, these disorders result in the progressive and discriminating loss of appropriate neuronal systems due to the accretion of misfolded proteins [2]. The polyglutamine (poly Q) tract generated by the CAG nucleotide triplet repeat induces polyglutamine-induced protein aggregation, which is a cornerstone of Huntington’s disease (HD) [3]. The previous line of research also demonstrated that the HD gene is located on chromosomal 4p16 and expresses huntingtin a 348 kDa glycoprotein that is abundantly distributed throughout synapses [4]. Excitotoxicity, energy deprivation, oxidative damage, inflammatory response, and protein expression are among the mechanisms through which neurodegeneration proceeds in HD [5].

By oxidative phosphorylation, mitochondria also produce adenosine triphosphate (ATP) and are the engine of the cell. They have a vital function in oxidative regulation, and program cell death, in addition to producing ATP [6]. Each impairment in mitochondrial biogenesis results in a variety of ailments. Numerous pieces of scientific evidence, including animal and human research, indicate that mitochondria play an important role in metabolic conditions such as diabetes, and neurologic illnesses such as HD, Alzheimer’s disease (AD), and Parkinson’s disease (PD) [6]. Previous data revealed how mitochondrial alteration relates to delayed neurodegeneration. Nevertheless, the molecular and physiological alterations that occur as neurodegenerative disorders advance are still not known [7].

Neurotoxin generated 3-Nitropropionic acid (3-NPA) from a complexioned milky vetch which is known to hinder the mitochondrial complex, resulting in deficiencies in the cellular components [6,7,8]. Since it impedes succinate dehydrogenase (SDH), it causes both striatal and oxidative responses similar to HD, which is an inherited disease that is typically expressed within the fourth decade of life [9,10]. The neurotoxic potential is induced via oxidative damage triggered by cellular energy loss. According to research, reactive oxygen species are among the primary sources of neuronal cell death in neurological conditions [11,12,13,14]. Several studies have found oxidative stress biomarker changes associated with HD [15,16,17]. 3-NPA-induced neurodegeneration is a prominent experimental model often employed to investigate the pathophysiology of HD [18]. In humans, 3-NPA-induced neurodegeneration is linked to immediate encephalitis, which is accompanied mostly by the emergence of delayed-onset dementia and locomotor defects [19].

Earlier studies hypothesized the significance of oxidative impairment in 3-NP-induced behavior, metabolic, and molecular impairment in rodents, as well as its putative amelioration by a substance of natural origin such as *Withania somnifera.* Notably, inflammation induced by microglial expressions plays a critical role in neurodegeneration such as seen in PD [20], AD [21], and HD [22]. Inflammatory mediators-induced neurodegeneration is triggered in part by prolonged and uncontrolled stimulation of microglia, as well as the enormous synthesis of inflammatory cytokines [23,24]. Among the primary inflammatory agents that cause the death of neurons is nitric oxide (NO), generated in microglia through the enzyme nitric oxide synthase (iNOS) [25]. Accordingly, inhibiting iNOS diminished microglial overexpression in neurodegeneration, demonstrating the importance of levels of NO in microglia-associated neurodegeneration [26].

**Figure 1:**
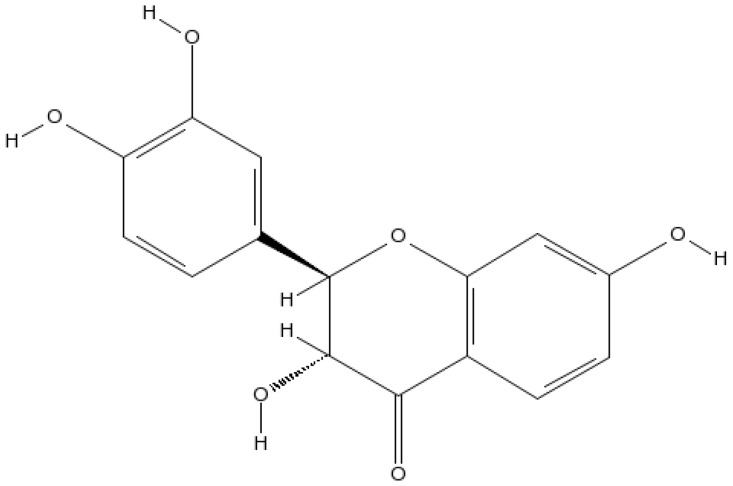
Fustin Structure.

A cofactor of tyrosine hydroxylase and phenylalanine hydroxylase, tetrahydrobiopterin (BH4) is needed for NOS activity [27]. Molecular oxygen and tetrahydrobiopterin are used by tyrosine hydroxylase to acclimatize tyrosine into levodopa (L-DOPA) [27]. This enzyme is the first step in BH4 synthesis and is the rate-determining enzyme [28]. In recent years, alternatives to conventional treatments with minimal side effects have gained increasing attention. Diverse natural compounds are appropriate for providing neuroprotective effects from neurodegeneration because of their accessibility, minimal price, better safety tolerances, and broad selection of therapeutic potential [29]. There have been several epidemiological studies focusing on flavonoids’ possible benefits for neurodegenerative diseases among the wide variety of plant-derived secondary metabolites. According to the amount of oxidation of the central C ring, hydroxylation pattern, and substitution at position 3 of the rings, there are over 5000 different flavonoids (flavones, flavanols, flavanones, anthocyanidins, and isoflavones). Glycosylation or alkylation are two ways of creating diversity within any group [30]. *Toxicodendron vernicifluum* (Stokes) F.A. Barkley (syn. *Rhus verniciflua* or *vernicifera* Stokes, Anacardiaceae), the lacquer tree, also known as sumac and heartwood of *Rhus verniciflua*, contains fustin (Figure 1), an active component [31]. Heartwood is not allergenic and has traditionally been used to treat rheumatoid arthritis and as a food additive. Researchers have recently discovered that *Rhus verniciflua* heartwood, which encompasses fustin, has anti-mutagenic, anti-arthritis, and antidiabetic properties mediated by antioxidants [32,33,34,35]. The findings of previous studies advise that fustin is neuroprotective against 6-OHDA-induced cell death and streptozotocin-induced cognitive impairment [36,37]. Fustin’s C-ring contains no double bonds [(2R,3R)-3,3′,4′,7-Tetrahydroxyflavan-4-one]. Fustin has two stereocenters and four stereoisomers, making it a flavan. *Acacia vestita*, *Acacia carneorum*, and other organisms contain fustin as a natural product. This study aims to determine if fustin can inhibit the development of Huntington’s disease in rodents induced by 3-NPA.

## 2. Methods

### 2.1. Animals

In the current protocol, male adult Wistar rats (220–280 gm body weight) were employed (*n* = 6). Animals were kept in the rodent care facilities under conventional lab settings including room temperature, humidity, and a 12:12 h light/dark cycle. Animals were provided with unrestricted food and water access. The present investigational method was authorized by the Animal Ethical Board (IAEC/TRS/PT/022/016), and all studies are carried out ensuring they met the criteria of the CPCSEA, Govt. of India.

### 2.2. Chemicals

The following chemicals were employed in the study: 3-NPA, malondialdehyde (MDA), succinate dehydrogenase (SDH), catalase (CAT), glutathione-S-transferase (GST), reduced glutathione (GSH), brain-derived natriuretic factor (BDNF), interleukins (IL-1β), tumor necrosis factor (TNF-α) and cyclooxygenase (COX) (MyBioSource, San Diego, CA, USA), measurement kit ATP estimate kit (Sigma-Aldrich, Inc., St. Louis, MO, USA), and fustin (>98%, stability ≥ 4 years, SKL, Mumbai, Maharashtra, India).

### 2.3. Experimental Design

The planned examination was based on previously reported data with minor adjustments. For the present study, rodents were categorized into five clusters *n* = 6. Normal saline (cluster-I), 3-NPA control (cluster-II), 3-NPA + fustin 100 mg/kg (cluster-III), and 3-NPA + fustin 50 mg/kg (cluster-IV), and fustin 100 mg/kg per se soluble in dimethyl sulfoxide (cluster-V). Initially, entire rats were exposed to acclimatization under standard laboratory settings for one week. For the quick induction of neurotoxicity in rodents, 3-NPA (10 mg/kg., i.p.) in saline was administered once daily for 15 days based on earlier reported data [38,39].

Similarly, during the 8th to 22nd days, the normal saline group received 3 mL/kg of saline, while the control group (3-NPA) received 3 mL/kg oral 0.5% sodium carboxymethyl cellulose 1 h after 3-NPA (10 mg/kg i.p) administration. The test group (50 mg/kg p.o.), and control group (100 mg/kg p.o.) received fustin. During the study, 1 h after the oral administrations, clusters II, III, and IV were given 3-NPA 10 mg/kg (i.p.). Throughout the experimental scheme, behavioral investigations were performed on the rodents. The rats lacked homogenate in their brain tissue on the last day of the experiment. The established homogenate was employed for the assessment of many biological components, comprising oxidative stress, endogenous (enzymatic and non-enzymatic activity) antioxidants, pro-inflammatory markers, neurotransmitter levels, and complex-II activity in the brain.

### 2.4. Behavioural Assessment

#### 2.4.1. Narrow Beam Walking Test

In this paradigm, a short beam was joined by a circular steel rod (0.5 mm × 2.0 cm × 120 cm). The beam was lifted 1 m just above the base floor. A sawdust-filled box was placed beneath the beam to soften the landing for the rats. Animals were given 5 min to acclimatize to their unfamiliar setting before starting training. The number of slips and the time it would require a rat to travel the beams from one side to another was documented in every experiment. The value was established by combining the three measured transfer latencies and the inter-trial duration lasting 2 min [40].

#### 2.4.2. Grip Strength Test

Gripping a horizontally tied cable latency was regarded as an excellent indicator of muscular gripping strength. Each rodent could hang by utilizing its forepaws on a 2 mm × 35 metal wire that was stretched horizontally at a maximum of 50 cm above a padded base. The period spent by each rodent holding the cable while immobile was documented [41].

#### 2.4.3. Rotarod Workout

Motor synchronization, grip strength, and rodent’s integrity were assessed at weekly intervals using a rotarod apparatus, similar to previously published data with slight modification [40,42]. The rotarod equipment is composed of four equal portions of a non-slippery shaft. To acclimate each animal to the apparatus, former training was employed. During the training, the rats were put on a 7 cm-diameter rotating shaft in both tests which was maintained at a speed of 25 rpm/min. To assess drop-down latency, a duration restriction of 180 s was put in place [43,44].

### 2.5. Biochemical Assessment

#### 2.5.1. Preparation of Homogenate

After decapitating the animals, the brain was removed and rinsed through isotonic saline (ice-cold). Different brain sections from individual animals (cortex, striatum, and hippocampus) were homogenized in phosphate buffer (ice-cold) (0.1 M, pH 7.4). Post-homogenization the obtained homogenate was subjected to centrifugation followed by biochemical estimation by employing supernatant [45,46].

#### 2.5.2. Brain Oxidative Parameters

##### Lipid Peroxidation and Endogenous (Enzymatic and Non-Enzymatic) Antioxidants

The production of MDA through the process of lipid peroxidation is an indicator of cellular damage. We assessed cellular damage in this experiment by detecting MDA levels within brain tissue homogenate. Previously, a tissue sample (0.1 mL) was mixed with a thiobarbituric acid reaction mixture (2 mL), which included 0.67% solution (1 mL) of thiobarbituric acid and 10% trichloroacetic acid solution (1 mL). Afterwards, the set reaction mixture was boiled for 30 min in a water bath, then cooled for 10 min in ice-cold water. Following cooling, the resultant mixture was subjected to centrifuge for ten minutes at (4830× *g*); the resultant supernatant was put forward for absorbance which was measured at 532 nm on a UV spectrophotometer (Shimadzu, Mumbai, Maharashtra, India). The assessment data were analyzed using previously reported data, and by employing an extinction coefficient of 1.56 × 105 M^−1^ cm^−1^ expressed in nmol MDA/mg wet mass of tissues [47].

Using slight adjustments, GSH was calculated using the data available. After mixing homogenate and trichloroacetic acid (10%) with identical volumes, 1 mL of supernatant was separated, and the 5,5′-dithio-bis-2-nitrobenzoic acid (DTNB) reagent (0.5 mL) was added to phosphate buffer (3 mL 0.2 M, pH 8) and 0.5 mL of DTNB. Afterwards, the absorbance at 412 nm of the miscellaneous solutions was calculated. The values were obtained by employing an extinction coefficient of 1.36 × 103 M^−1^ cm^−1^ and are reported in nmol GSH/mg wet weight of tissue [48].

CAT enzymes reduce oxidative stress in tissues by decomposing hydrogen peroxide (H_2_O_2_). CAT was measured using the Claiborne (1985) technique, with slight changes. 0.5 M phosphate buffer (pH 7) was mixed with 30 mM H_2_O_2_ (1 mL) per ml to obtain the results. This chemical process was initiated by the addition of 0.1 mL of the homogenate. A spectrophotometric measurement at 240 nm was conducted to measure the reduction in absorbance rate caused by the breakdown of H_2_O_2_. An extinction coefficient of 43.6 M^−1^ cm^−1^ was cast off to compute CAT activity and it was expressed as micromole per gram of wet tissue mass [49].

The amount of SOD was determined using the previously reported method [50].

#### 2.5.3. SDH (Complex-II)

In the process of transforming succinic acid into fumaric acid, potassium ferricyanide is used as an electron acceptor. In the present experiment, 0.05 mL mitochondrial suspension was mixed with 0.2 M, 1.5 mL phosphate buffer (pH 7.8), 0.6 M, 0.2 mL succinic acid (pH 7.8), and 0.3 mL BSA (1% *w*/*v*). Water was used as a control to measure succinate dehydrogenase by measuring a decrease in absorbance at 420 nm for 3 min. SDH expressed as in nmol/min/g [42].

#### 2.5.4. Enzyme-Linked Immunosorbent Assay

The appropriate brain slices were excised and homogenized to create 10% saline samples. Following that, centrifugation was undertaken, and the total protein content in the homogenates was evaluated as per previously reported data [51]. The homogenates were also employed to screen GST, BDNF, COX, IL-1β, TNF-α, GABA (gamma-aminobutyric acid), glutamate, and nitrate levels using several widely viable ELISA assays.

### 2.6. Statistical Analysis

To scrutinize the data concerns in the current investigation, we cast-off GraphPad Prism 5 (Dotmatics, Boston, MA, USA) for Windows. The outcomes were plotted as mean ± standard error mean (S.E.M.). A one-way analysis of variance (ANOVA) was undertaken, followed by Tukey’s multiple evaluation assessment, to investigate the degree of implication and display the deviation among the limits within an individual group. Statistical significance was defined as *p* value less than 0.05.

## 3. Results

### 3.1. Behavioural Analysis

#### 3.1.1. Effect of Fustin on Narrow Beam Walk Assessment

**Figure 2:**
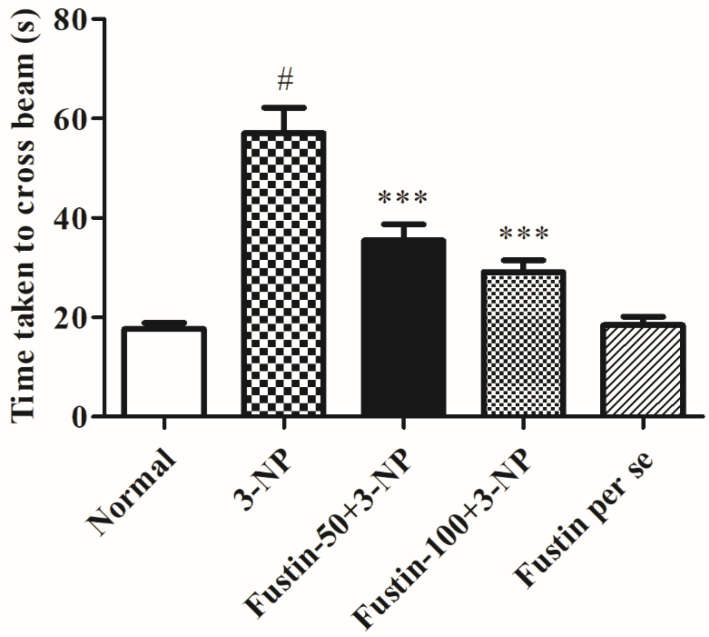
Effect of fustin on beam walk test against 3-nitropropionic acid-induced Huntington’s-like effects in rats. Values are expressed as mean ± S.E.M. (*n* = 6). Values are statistically significant at # *p* < 0.05 vs. negative control group, *** *p* < 0.001 vs. 3-nitropropionic acid, respectively (One-way ANOVA followed by Tukey’s test).

Figure 2 represents the outcome of fustin administration on the narrow beam walk behavioral assessment. The findings of the current research revealed that animals from the 3-NPA-control group took longer to traverse the wooden deck (*p* < 0.001) than rats in the conventional saline-treated group. Conversely, animals given a high dosage of fustin (100 mg/kg) reinstated typical cognitive performance by substantially decreasing the time necessary to walk across the beam (*p* < 0.001). Correspondingly, when associated with the 3-NPA-control, a small dosage of fustin (50 mg/kg) slightly reinstated typical cognitive performance by a marked decline in the time necessary to walk across the beam (*p* < 0.001). Nevertheless, one-way ANOVA (Tukey’s post hoc) parametric assessment discovered that the fustin group’s performance showed no effect on the time throughout beam walk factors in experimental animal models.

#### 3.1.2. Influence of Fustin on the Grip Strength Test

**Figure 3:**
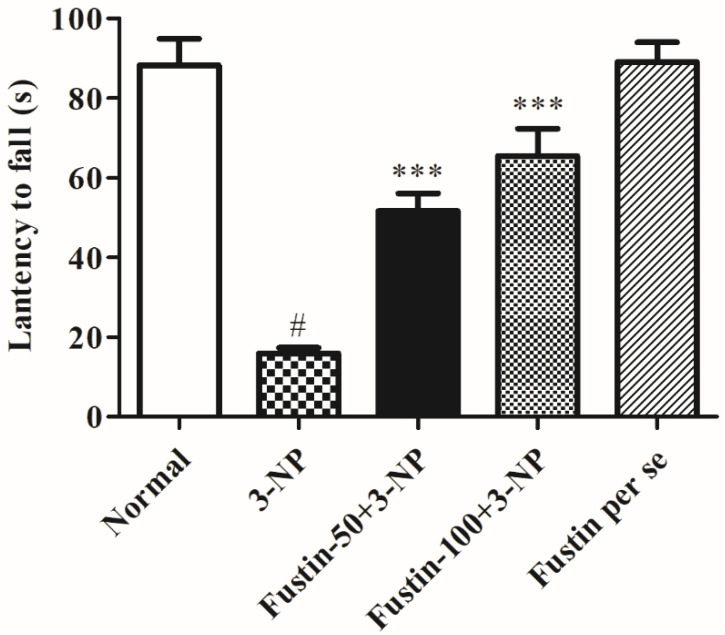
Effect of fustin on gripping strength against 3-nitropropionic acid-induced Huntington’s-like effects in rats. Values are expressed as mean ± S.E.M. (*n* = 6). Values are statistically significant at # *p* < 0.05 vs. negative control group, *** *p* < 0.001 vs. 3-nitropropionic acid, respectively (One-way ANOVA followed by Tukey’s test).

Figure 3 represents the outcome of fustin administration on the grip strength test behavioral assessment. The findings of the current research exposed that animals in the 3-NPA-control had a remarkably lower potential of falling (*p* < 0.001) than rats in the conventional saline-treated group as a hallmark of declined skeletal muscle activity. Conversely, animals given a high dosage of fustin (100 mg/kg) reinstated typical skeletal muscle strength, as seen by their improved time before falling (*p* < 0.001). Correspondingly, compared with 3-NPA-control group, a small dosage of fustin (50 mg/kg) moderately reinstated typical skeletal muscle strength, again indicated by improved time before falling (*p* < 0.01). Nevertheless, one-way ANOVA (Tukey’s post hoc) parametric assessment publicized that the fustin group had no effect on fall time in rats.

#### 3.1.3. Effect of Fustin on Rotarod Paradigm

**Figure 4:**
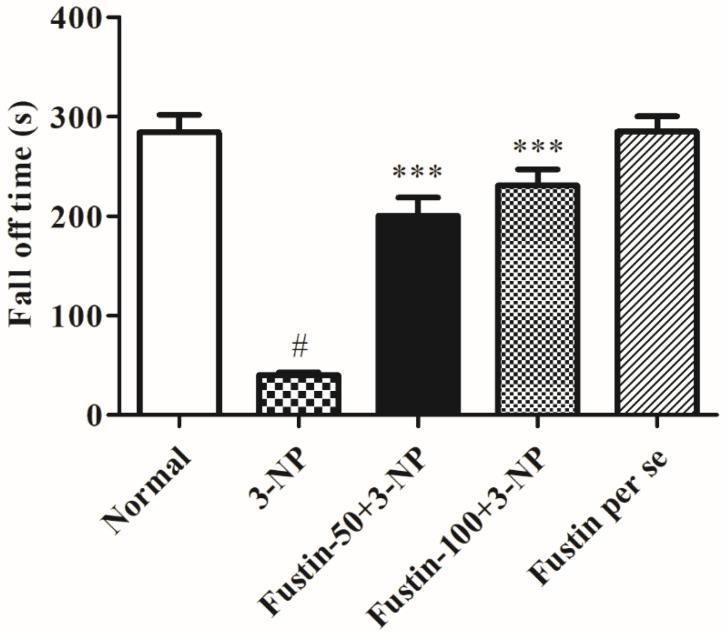
Effect of fustin on rotarod test against 3-nitropropionic acid-induced Huntington’s-like effects in rats. Values are expressed as mean ± S.E.M. (*n* = 6). Values are statistically significant at # *p* < 0.05 vs. negative control group, *** *p* < 0.001 vs. 3-nitropropionic acid, respectively (Two-way ANOVA followed by Tukey’s test).

Figure 4 symbolizes the consequence of fustin on a rotarod test evaluation. The findings of the current research shows that rats in the 3-NPA-control showed a significantly shorter fall time (*p* < 0.001) than rats in the conventional saline-treated group, a hallmark of declined skeletal muscular activity. Conversely, animals given a high dosage of fustin (100 mg/kg) reinstated typical skeletal muscle strength by significantly (*p* < 0.001) enhanced fall latency levels compared to the 3-NPA group. Correspondingly, when equated with 3-NPA-control, a small dosage of fustin (50 mg/kg) moderately reinstated typical skeletal muscle strength by enhanced fall off time duration (*p* < 0.001). Nevertheless, one-way ANOVA (Tukey’s post hoc) parametric assessment showed that the fustin group had no effect on the fall off time in rats. In the present investigation we evaluated its ability to interfere with normal muscle strength in rats, which was found to decrease significantly in the 3-NPA-treated group as compared with normal saline treatment. Simultaneously, we also evaluated the same effects in the per se treated group to rectify whether drug treatment alone had any interference with normal muscle activity in rats.

### 3.2. Biochemical Assessment

#### 3.2.1. Influence of Fustin on Brain Oxidative and Endogenous (Enzymatic and Non-Enzymatic) Antioxidants Parameters in Experimental Animal Models

**Figure 5:**
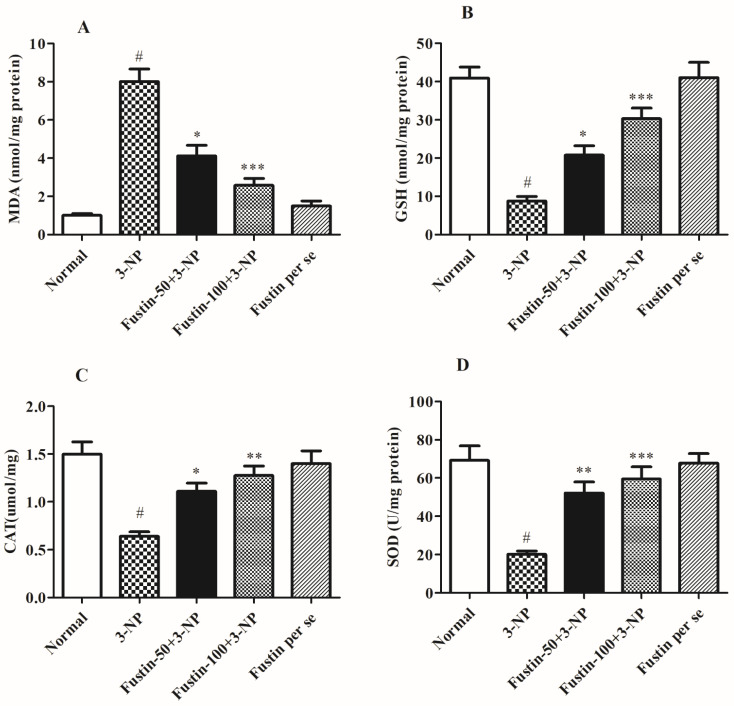
Effect of fustin on endogenous parameters against 3-nitropropionic acid-induced Huntington’s-like effects in rats (**A**) MDA, (**B**) GSH, (**C**) CAT, (**D**) SOD. Values are expressed as mean ± S.E.M. (*n* = 6). Values are statistically significant at # *p* < 0.05 vs. negative control group, * *p* < 0.05 vs. 3-nitropropionic acid, ** *p* < 0.01 vs. 3-nitropropionic acid, *** *p* < 0.001 vs. 3-nitropropionic acid, respectively (One-way ANOVA followed by Tukey’s test).

Figure 5A–D shows the consequence of fustin on several oxidative factors. In the assessment, when compared with the normal saline-treated animals, 3-NPA-induced animals exhibited considerable (*p* < 0.001) elevation in MDA activity as a crucial indicator for ROS. Equally, in an alternative set of experiments, 3-NPA-induced rats presented a considerable decrease in oxidative markers including CAT, GSH, and SOD in the brain perfusion (*p* < 0.001). Conversely, animals given a high dosage of fustin (100 mg/kg) showed a pointed decline in the increased MDA activity, and re-established reduced GSH, CAT, and SOD levels in the brain tissue perfusion (*p* < 0.001). Correspondingly, while related to the 3-NPA-control group, a small dosage of fustin (50 mg/kg) showed a less pointed (*p* < 0.05) decline in the increased MDA activity, and moderately re-established reduced GSH, CAT (*p* < 0.05), and SOD (*p* < 0.01) levels in the brain tissue perfusion. Nevertheless, one-way ANOVA (Tukey’s post hoc) parametric assessment publicized that the fustin group showed no effect on any of the above-mentioned biochemical paradigms in the rats.

#### 3.2.2. Influence of Fustin on Brain Pro-inflammatory Markers in Experimental Animal Models

**Figure 6:**
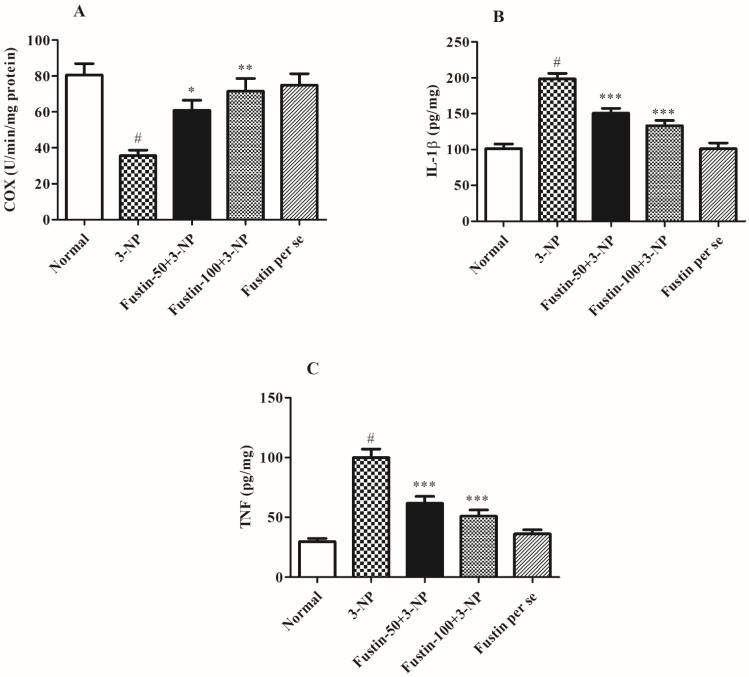
Effect of fustin on brain pro-inflammatory markers against 3-nitropropionic acid-induced Huntington’s-like effects in rats (**A**) COX, (**B**) IL-1β, (**C**) TNF-α. Values are expressed as mean ± S.E.M. (*n* = 6). Values are statistically significant at # *p* < 0.05 vs. negative control group, * *p* < 0.05 vs. 3-nitropropionic acid, ** *p* < 0.01 vs. 3-nitropropionic acid, *** *p* < 0.001 vs. 3-nitropropionic acid, respectively (One-way ANOVA followed by Tukey’s test).

Figure 6A–C shows the effect of fustin on several pro-inflammatory markers. In the analysis, compared with a normal saline-treated group, 3-NPA-induced animals had a highly substantial increase (*p* < 0.001) in IL-1β, TNF-α, and a significant decline in the COX level as a key biomarker for inflammatory responses occurred within the neuronal tissues. On the contrary, animals given a high dosage of fustin (100 mg/kg) showed a remarkable decline in the increased IL-1β, and TNF-α (*p* < 0.001), and a moderate decline in the COX level (*p* < 0.01) in the brain tissue perfusion. Correspondingly, when compared to the 3-NPA-control, a small dosage of fustin (50 mg/kg) showed a significant (*p* < 0.001) decline in the increased IL-1β, TNF-α, and a minimal re-establishment of the declined level of COX in the brain tissue perfusion (*p* < 0.05). Nevertheless, one-way ANOVA and Tukey’s post hoc parametric test exposed that the fustin group had no effect on any of the above-mentioned biochemical paradigms in the rats.

#### 3.2.3. Influence of Fustin on Enzymatic Activity in Experimental Animal Models

**Figure 7:**
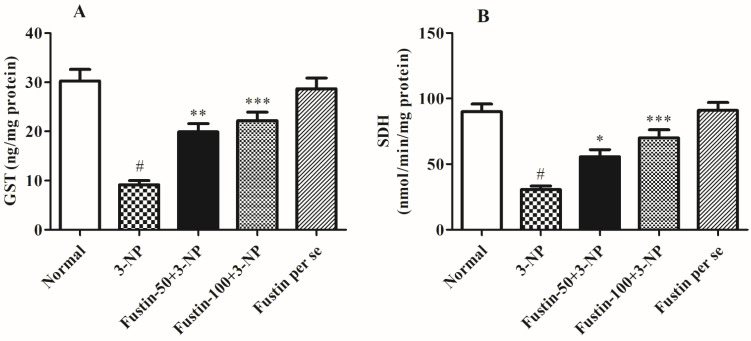
Effect of fustin on enzymatic activity against 3-nitropropionic acid-induced Huntington’s-like effects in rats (**A**) GST, (**B**) SDH. Values are expressed as mean ± S.E.M. (*n* = 6). Values are statistically significant at # *p* < 0.05 vs. negative control group, * *p* < 0.05 vs. 3-nitropropionic acid, ** *p* < 0.01 vs. 3-nitropropionic acid, *** *p* < 0.001 vs. 3-nitropropionic acid, respectively (One-way ANOVA followed by Tukey’s test).

Figure 7A,B shows the effect of fustin on several enzymatic activities. In the investigation, compared with a normal saline-treated group, 3-NPA-induced animals had a highly substantial decline (*p* < 0.001) in GST and SDH-II activity as a key biomarker for neurodegeneration within the brain tissues. On the contrary, animals given a high dosage of fustin (100 mg/kg) remarkably restored the decline in GST, and SDH-II activity (*p* < 0.001). While associated with the 3-NPA-group, a lower dosage of fustin (50 mg/kg) less significantly (*p* < 0.05), and more significantly (*p* < 0.001) restored the decline of the GST, and SDH-II activity, respectively. Nevertheless, one-way ANOVA and Tukey’s post hoc parametric assessment showed that the fustin group had no effect on any of the above-mentioned biochemical paradigms in the rats.

#### 3.2.4. Influence of Fustin on Neurotransmitter Levels in Experimental Animal Models

**Figure 8:**
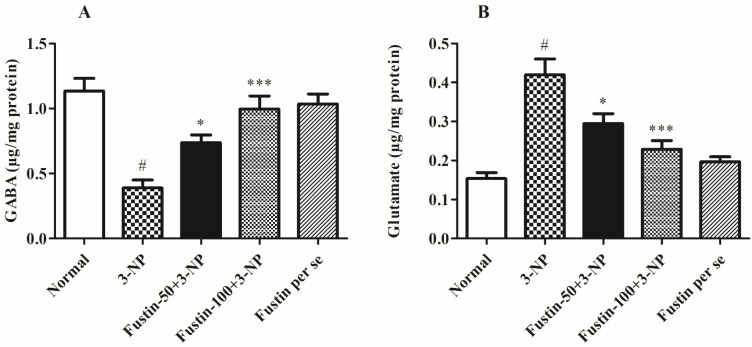
Effect of fustin on neurotransmitter levels against 3-nitropropionic acid-induced Huntington’s-like effects in rats (**A**) GABA, (**B**) Glutamate. Values are expressed as mean ± S.E.M. (*n* = 6). Values are statistically significant at # *p* < 0.05 vs. negative control group, * *p* < 0.05 vs. 3-nitropropionic acid, *** *p* < 0.001 vs. 3-nitropropionic acid, respectively (One-way ANOVA followed by Tukey’s test).

Figure 8A,B displays the effect of fustin on several enzymatic activities. In the investigation, compared with a normal saline-treated group, 3-NPA-induced animals had a considerably high decline (*p* < 0.001) in GABA level and an increased level of glutamate as a key biomarker for alteration in the normal neuronal activity. On the contrary, animals given a high dosage of fustin (100 mg/kg) had a remarkably restored level of GABA, and showed a decrease in the levels of previously raised glutamate (*p* < 0.001). Additionally, while associated with the 3-NPA-control group, a small dosage of fustin (50 mg/kg) less significantly (*p* < 0.05) restored the decline of GABA, and decreased the elevated levels of glutamate, respectively. Nevertheless, one-way ANOVA (Tukey’s post hoc) parametric assessment showed that the fustin group had no effect on any of the levels of the above-mentioned neurotransmitter in the rats.

#### 3.2.5. Influence of Fustin on BDNF Activity in Experimental Animal Models

**Figure 9:**
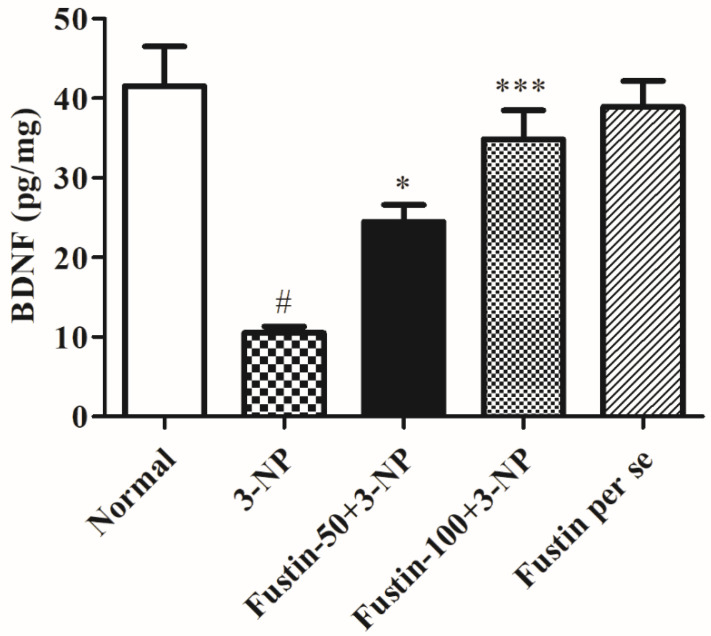
Effect of fustin on BDNF activity against 3-nitropropionic acid-induced Huntington’s-like effects in rats. Values are expressed as mean ± S.E.M. (*n* = 6). Values are statistically significant at # *p* < 0.05 vs. negative control group, * *p* < 0.05 vs. 3-nitropropionic acid, *** *p* < 0.001 vs. 3-nitropropionic acid, respectively (One-way ANOVA followed by Tukey’s test).

Figure 9 displays the effect of fustin on BDNF activity. In the investigation, compared with the normal saline-treated group, 3-NPA-induced animals had a considerably high depletion in the brain BDNF activity (*p* < 0.001) which signifies potency of 3-NPA in denaturation of the brain and neuronal tissues. We found that one-way ANOVA (Tukey’s post hoc) parametric analysis exposed a daily high dose of fustin (100 mg/kg) treatment resulted in a significant variation in BDNF activity (*p* < 0.001), whereas a low dose of fustin showed minimal decline in the brain BDNF activity (*p* < 0.05) when associated with the 3-NPA-control in rats.

#### 3.2.6. Influence of Fustin on Nitrite Level in Experimental Animal Models

**Figure 10:**
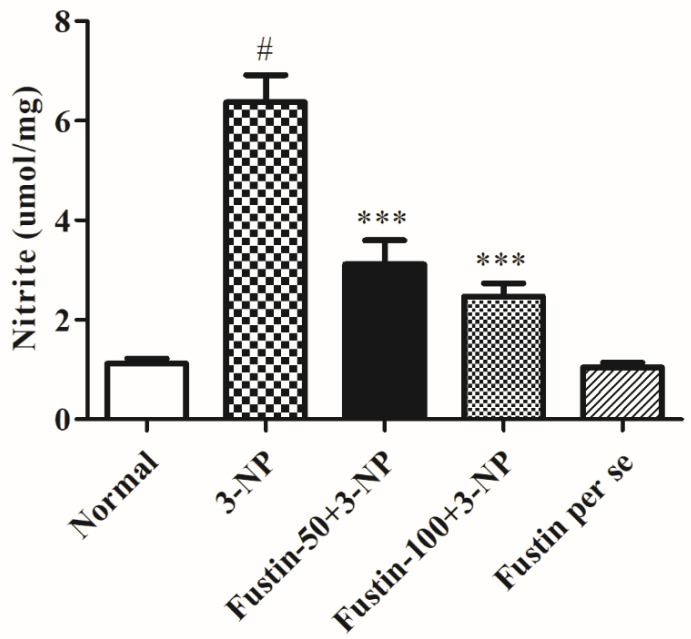
Effect of fustin nitrite level against 3-nitropropionic acid-induced Huntington’s-like effects in rats. Values are expressed as mean ± S.E.M. (*n* = 6). Values are statistically significant at # *p* < 0.05 vs. negative control group, *** *p* < 0.001 vs. 3-nitropropionic acid, respectively (One-way ANOVA followed by Tukey’s test).

Figure 10 depicts the outcome of fustin on nitrite levels in the brain tissue. In the study, compared with the normal saline-treated group, 3-NPA-induced animals had a considerable rise in their nitrite levels (*p* < 0.001), which signifies the potency of 3-NPA in its alteration of the brain and neuronal activities. In our findings, one-way ANOVA (Tukey’s post hoc) parametric analysis revealed that daily low and high doses of fustin (100 mg/kg) treatment resulted in a significant decline in the nitrite level (*p* < 0.001), whereas fustin showed no clinical alteration in the nitrite level when associated with the 3-NPA-control in rats.

## 4. Discussion

Fustin was investigated as a possible neuroprotective drug towards 3-NPA-induced neurotoxicity in rodents in the present investigation for HD. The preceding study has demonstrated the hazardous capability of 3-NPA, a mitochondrial toxic which triggers HD-like manifestations [18]. In this study, we discovered that 3-NPA caused severe HD-like characteristics in experimental animals. Multiple experimental studies revealed that 3-NPA treatment had a significant impact on mouse locomotor skills [52]. Moreover, we evaluated standard control rodents with 3-NPA-controlled rodents in our investigation and discovered that 3-NPA-treated rats experienced significant changes, such as a significant decline in several behavioral and biochemical parameters. Furthermore, during the testing we found that animals treated with both doses (50 mg, and 100 mg/kg) of fustin successfully restored their cognitive performance as compared with the control-treated group, under assessment of several behavioral paradigms including the narrow beam walk assessment, grip strength test, and rotarod paradigm. Results reveal that alteration in behavioral and biochemical parameters is a key aspect of HD [53].

In our present research, we included multiple biochemical estimates to highlight changes related to HD in preclinical animals. The primary focus of laboratory tests was on oxidative indicators, inflammatory component measurement, changes in various specific enzymes, neurotransmitter measurement, and protein analysis. Prior studies looked at the role of many oxidative markers in the etiology of HD, with a focus on CAT, GSH, GR, SOD, LPO, and GPx in the hippocampus and neural cells. Furthermore, investigations have shown a significant decrease in the levels of numerous indicators such as CAT, GSH, GR, and SOD in 3-NPA-generated rodent models for HD [54,55]. As in the current study, 3-NPA-treated rats have considerably lower degrees of CAT, GSH, and SOD in the rat brain, as well as higher amounts of MDA. Using fustin for a subsequent 15 days post-inducement substantially maintained the above-mentioned biomarkers, indicating that fustin is a prospective neuroprotective agent in HD-like manifestations.

Interestingly, similar research found that a decrease in the level of neurotransmitters was apparent throughout chemical-induced neurotoxicity. Moreover, serotonin, norepinephrine, dopamine, GABA, and glutamate have also been recognized as crucial candidates in the pathology of HD [56,57,58,59]. We examined the quantities of different monoamine neurotransmitters and complementary amino acids in tissue homogenate in this investigation. Further, in our investigation we saw that glutamate and GABA levels were drastically altered by the infusion of 3-NPA. On the contrary, fustin therapy enhances the above indicators greatly in experimental animals.

Earlier studies have revealed that BDNF and nitrite levels are lower in HD experimental animals [60]. Furthermore, this drop is attributable to ROS-increased production, BDNF mRNA downregulation, and alteration of huntingtin protein expression [61]. Likewise, we discovered that rodents treated with 3-NPA have substantial changes in BDNF and aberrant nitrite levels. Furthermore, pre-treatment with fustin greatly reverses the above-mentioned nitrite downregulations and enhanced levels of BDNF, indicating a neurodefensive effect in 3-NPA-induced neurotoxicity in rodents. Numerous studies show that neurotoxicity is linked to significant increases in pro-inflammatory level mediators, including TNF-α, COX and IL-1β [40,62]. In our investigation, we discovered that 3-NPA infusion significantly impacted the concentrations of each of the abovementioned indicators that were substantially decreased by fustin injection, indicating the anti-neuroinflammatory activity of fustin mediated via antioxidant activity. These findings demonstrated that fustin, a flavonoid, may also contribute to neuroprotective effects via improvement in behavioral activities, endogenous (enzymatic and non-enzymatic) antioxidant function and also reduces the concentration of neurotoxins by preventing oxidative stress, maintained the BDNF, GST, SDH-II complexes, nitrite levels, TNF-α, IL-1β, COX, GABA, and glutamate level in the brain. To the best of our knowledge, this is the first report on the use of a novel phytoconstituent fustin that offered neuroprotection by subsiding enzymatic and oxidative activity in the 3-NPA-induced HD experimental animals paradigm. The limitations of the study are its short duration and the use of minimal animals. The present investigation aimed to find out the neuroprotective efficiency of fustin, but further molecular studies are required to elaborate its specific potential on a molecular basis to serve more works in the near future and confirm our findings related to the cognitive recovery from fustin treatment. In addition, higher doses of fustin could be tested in preclinical and clinical studies for HD.

## 5. Conclusions

The present study shows the first appealing use of fustin as an effective neuroprotectant, with the capability to progress some biochemical markers of redox damage and inflammation, as well as boost the altered neurotransmitter in 3-NPA-induced neurotoxicity in experimental animals. Moreover, we showed that fustin exhibits neuroprotective properties via downregulating the various protein biomarkers such as BDNF, SDH-II complexes, and nitrite levels. Interestingly, fustin was also found to be protective against HD-like symptoms in this model via regulating monoaminergic neurotransmission. This could result in the creation of low-cost flavonoid alternatives for HD treatment. The current findings of the study revealed that fustin has the possibility to be a useful natural constituent in the management of neurological illnesses such as HD.

## Data Availability

Not applicable.
